# Correlation of Liver Elastography as a Predictor of Esophageal Varices and Its Comparison With Ultrasound Abdomen and Liver Function Tests in Patients With Chronic Liver Disease

**DOI:** 10.7759/cureus.41652

**Published:** 2023-07-10

**Authors:** Naveen AS, Suja Lakshmanan, N Senthil, Varsha R

**Affiliations:** 1 Internal Medicine, Royal Care Hospital, Coimbatore, IND; 2 Internal Medicine, Sri Ramachandra Institute of Higher Education and Research, Chennai, IND; 3 General Medicine, Sri Ramachandra Institute of Higher Education and Research, Chennai, IND; 4 General Medicine, Dr. Kamakshi Memorial Hospital, Chennai, IND

**Keywords:** liver scores, non-invasive predictors, liver stiffness, liver elastography, cirrhosis, oesophageal varices

## Abstract

Introduction: Variceal haemorrhage is a life-threatening complication that occurs in up to 40% of patients with chronic liver disease including cirrhosis. It is associated with a mortality rate of 20% with each episode of variceal bleeding. Esophagogastroduodenoscopy is the gold standard for the detection of esophageal varices but is an invasive procedure and not very cost-effective. Our study was designed to correlate the presence of esophageal varices on endoscopy with the liver stiffness measurement using liver elastography in patients with chronic liver disease. We also compared various non-invasive predictors like laboratory parameters and ultrasound features and correlated them with the presence of varices in patients with chronic liver disease.

Methodology: This prospective observational study was conducted in a tertiary-care hospital in South India from November 2017 to April 2019. All patients with chronic liver disease were subjected to endoscopy, and the presence of esophageal varices and their grading was noted. The predictive efficacy of ultrasound elastography using Toshiba Aplio 500 ultrasound two-dimensional shear wave elastography (2D-SWE) in predicting esophageal varices was calculated and compared with the efficacy of predicting esophageal varices by other non-invasive parameters like laboratory investigations, abdominal ultrasound, and liver scores like Child-Turcotte-Pugh (CTP) score, model for end-stage liver disease (MELD) score, fibrosis 4 (FIB-4) score, aspartate aminotransferase-to-platelet ratio index (APRI) score, and aspartate aminotransferase/alanine aminotransferase ratio (AAR).

Results: The study included a total of 168 patients out of which 57% (96 patients) had no varices. About 52 patients (72.2%) had F1/Grade I varices, 9 (12.5%) had F2/Grade II varices, and 11 (15.3%) had F3/Grade III varices. The greatest predictive value for esophageal varices was liver stiffness with a diagnostic accuracy of 81.7%. Ultrasound features like coarse echotexture of the liver (66.7%), splenomegaly (67%), dilated portal vein (78.6%), and presence of moderate ascites (66.7%) had a significant statistical association with the presence of esophageal varices. Laboratory parameters like thrombocytopenia of less than 1.5 lakhs/cu.mm (52.8%), albumin <3 g/dL (60.4%), and reversal of albumin/globulin ratio (52.4%) were significant predictors of esophageal varices. The odds ratio for significant scores in predicting oesophageal varices using binary logistic regression was significant in patients whose liver elastography grade was more than F4, CTP score was B, MELD score was >11, and FIB-4 scores was >3.25 and between 1.46 and 3.25.

Conclusion: Liver elastography is a non-invasive procedure that can be a useful tool in predicting esophageal varices in chronic liver disease. Other non-invasive predictors like ultrasound abdomen and laboratory parameters can also be considered a replacement for repeated invasive endoscopy, thus facilitating early intervention and avoiding unfavourable outcomes in patients with chronic liver disease.

## Introduction

Chronic liver disease involves progressive destruction and regeneration of the liver parenchyma eventually leading to fibrosis and cirrhosis causing decompensated liver function. The most common complication of chronic liver disease is portal hypertension featuring its complications with a triad of gastroesophageal varices, ascites, and splenomegaly. Variceal haemorrhage is a life-threatening complication that occurs in 25% to 40% of patients with cirrhosis [1] with a 20% mortality rate associated with every episode of bleeding [2-5]. The grade of esophageal varices often correlates with the severity of liver disease. In a patient with an acute episode of variceal haemorrhage, there is a 70% greater risk of recurrent bleeding within the same year [6]. Hence, early suspicion and screening for the presence of varices is the cornerstone in the management of portal hypertension.

Esophagogastroduodenoscopy is the gold standard for the detection of esophageal varices. The progression of GI varices is determined by their size classification [7]. Endoscopy is an invasive procedure and not very cost-effective [8]. Cost-effectiveness can be overcome if endoscopy is performed only in high-risk groups. Many factors including clinical, laboratory, and ultrasound parameters either alone or in combination have been studied as a predictive tool for the presence of esophageal varices and assessing the risk of variceal haemorrhage including low platelet count, splenic diameter, platelet count to spleen diameter ratio, portal vein size or presence of collaterals on ultrasound, and hypersplenism [9-11]. All these studies were mainly done to select the high-risk group with esophageal varices so that endoscopy can be done in selected patients, thereby avoiding unnecessary intervention or financial burden but not missing the patients at risk of bleeding.

Our study was designed to study the liver stiffness measurement using elastography and to correlate with the presence of esophageal varices on upper GI endoscopy in patients with chronic liver disease. This study also compared various laboratory and radiological parameters in chronic liver disease patients that correlate with the presence of esophageal varices on upper GI endoscopy. The study also analysed the parameters that will enable early prediction of the presence of esophageal varices in at-risk patients, thereby eliminating unwarranted endoscopic procedures.

## Materials and methods

This prospective observational study was conducted in a tertiary-care hospital from November 2017 to April 2019. Patients with chronic liver disease as defined by the study methodology were included in the study. The subjects included had progressive deterioration of liver functions for more than six months duration. Subjects with features of chronic liver disease on ultrasound abdomen and liver elastography were also part of the study. The selected patients were either admitted to the medical or medical gastroenterology wards. We also included patients who were treated as outpatients after obtaining appropriate consent. All the eligible subjects were recruited consecutively by convenient sampling until the end of the study period. Upper GI endoscopy was performed in all patients to look for the presence of esophageal varices. Patients less than 18 years of age, patients with active upper GI bleeding other than esophageal varices, and patients who have undergone endoscopic or surgical intervention for the management of esophageal varices were excluded from the study. Patients with aspartate aminotransferase (AST) or alanine aminotransferase (ALT) greater than 100 IU/L and patients with ascitic fluid greater than 1500 ml were also excluded. The study was approved by the institutional ethics committee (CSP-MED/17/N0V/40/151). Informed consent was obtained from all the study participants. The study participants were assessed for detailed history, comorbid conditions, and clinical examination.

Laboratory investigations including complete blood count, renal function test, liver function tests, coagulation and lipid profile, serum electrolytes, and viral markers were obtained. Specific tests like serum antinuclear antibody, anti-smooth muscle antibody, liver-kidney microsomal type1 antibody, serum ceruloplasmin, and urinary copper, if done as part of the evaluation of etiology in selected patients, were also included. Ascitic fluid analysis and calculation of serum-ascites albumin gradient if done were obtained. Radiological data comprising liver and spleen size, echo texture of the liver, size of the portal vein and splenic vein, and the presence of free fluid were obtained by using USG. CT of the abdomen was performed in patients if warranted. Liver elasticity using two-dimensional shear wave elastography (2D-SWE) using acoustic radiation force impulse (ARFI) was done. The patients were grouped into different grades of liver fibrosis according to the elasticity values measured in kPa based on their etiology. Using relevant parameters from the data collected, the Child-Turcotte-Pugh (CTP), model for end-stage liver disease (MELD) score, and fibrosis 4 (FIB-4) score were calculated. The upper limit of AST was fixed as 50 IU/L for males and 35 IU/L for females for calculating ratios like AST-to-platelet ratio index (APRI) and AST/ALT ratio (AAR). The presence of esophageal varices in different grades of liver fibrosis measured by elastography was noted, and the predictive efficacy of ultrasound elastography was calculated and compared with the efficacy of prediction of esophageal varices by other non-invasive parameters and scores.

The data collected were analysed using SPSS Statistics version 22.0 (IBM Corp. Released 2011. IBM SPSS Statistics for Windows, Version 20.0. Armonk, NY: IBM Corp.). Descriptive statistics such as mean and standard deviation (SD) for continuous variables, frequencies, and percentages were calculated for categorical variables. To check for associated factors with the outcome, the Chi-square test and Mann-Whitney U test were used. In the case of lesser expected counts, Fisher exact test was used. To find the strength of the association (odds ratio) of factors with the outcome, binary logistic regression was used. To determine the best cut-off of the scores in predicting outcome, the receiver operator characteristics curve was used. Bar and pie charts were used for a visual representation of data. The level of significance was set at 0.05.

## Results

A total of 187 patients were screened for chronic liver disease and enrolled from November 2017 to April 2019. Later, 19 patients were excluded based on the exclusion criteria. The total number of patients who were included in this study was 168. The demographic characteristics of the study participants are shown in Table 1.

**Table 1:** Demography and clinical profile and its association with esophageal varices in the study cohorts NASH: nonalcoholic steatohepatitis

Parameters	Varices n (%)			Total		p-value
	Absent	Present				
1. Age in years						
18-30	14 (82.4%)	3 (17.6%)		17 (10%)		<0.001
31-45	39 (73.6%)	14 (26.4%)		53 (31.5%)		
46-60	34 (54.8%)	28 (45.2%)		62 (37%)		
>60	9 (25%)	27 (75%)		36 (21.5%)		
Total	96 (57%)	72 (43%)		168 (100%)		
2. Gender						
Male	72 (58.5%)	51 (41.5%)		123 (73.2%)		0.546
Female	24 (53.3%)	21 (46.7%)		45 (26.8%)		
Total	96 (57%)	72 (43%)		168 (100%)		
3. Comorbid condition						
Diabetes mellitus						
Present	23 (33.8%)		45 (66.2%)	68 (40.5%)		<0.001
Absent	73 (73%)		27 (27%)	100 (59.5%)		
Total	96 (57%)		72 (43%)	168 (100%)		
4. Hypertension						
Present	15 (38.5%)		24 (61.5%)		39 (23.2%)	0.007
Absent	81 (62.7%)		48 (37.3%)		129 (76.8%)	
Total	96 (57%)		72 (43%)		168(100%)	
5. Dyslipidaemia						
Present	17 (54.8%)	14 (45.2%)		31 (18.5%)		0.774
Absent	79 (57.7%)	58 (42.3%)		137 (81.5%)		
Total	96 (57%)	72 (43%)		168 (100%)		
6. Hypothyroidism						
Present	9 (50%)	9 (50%)		18 (10.7%)		0.516
Absent	87 (58%)	63 (42%)		150 (89.3%)		
Total	96 (57%)	72 (43%)		168(100%)		
7. Etiology of liver disease						
Alcohol	39 (57.4%)	29 (42.6%)		68 (40.5%)		0.573
NASH	16 (48.5%)	17 (51.5%)		33 (19.6%)		
Unknown	26 (63.5%)	15 (36.5%)		41 (24.4%)		
Hepatitis B	9 (69.3%)	4 (30.7%)		13 (7.8%)		
Hepatitis C	4 (57.2%)	3 (42.8%)		7 (4.2%)		
Others (hepatocellular carcinoma, autoimmune hepatitis, Wilson disease, tuberculosis with non-cirrhotic portal fibrosis)	2 (33.4%)	4 (66.6%)		6 (3.5%)		
Total	96 (57%)	72 (43%)		168 (100%)		

The median age of the study population was 48 years with a range from 19 to 85 years. The presence of esophageal varices was 75% in the age group of >65 years followed by 45.2% in the age group of 46-60 years and 26.4% in the age group of 31-45 years. There was a significant association of age with the presence of varices with a p-value of <0.001. Males (73.2%) outnumbered females (26.8%). Varices were present in 41.5% of the males and 46.7% of the females. There was no significant association of gender with varices with a p-value of 0.546.

The clinical profile of the subjects and its association with esophageal varices is depicted in Table 1. Diabetes was found in 40.5% (n= 68) of the study population. The distribution of varices among the diabetic population was 66.2% (n=45) and among the non-diabetic patients was 27%. Hypertension was seen in 23.2% of the participants (n=39) out of which 61.5% (n=24), had varices and only 37.3% without hypertension had varices. There was no significant association between the presence of varices and dyslipidemia (p=0.774) or hypothyroidism (p= 0.516) (Table 1). The various causes of cirrhosis in our study population are depicted in Table 1. Varices were seen in the majority of the people with alcohol- (42.6%) and NASH-related (51.5%) cirrhosis followed by 42.8% in cirrhosis related to hepatitis C and 30.7% in hepatitis B infection. About 66.6% of patients with other causes had varices. However, there was no significant association between different etiologies and varices (p=0.573).

**Figure 1:**
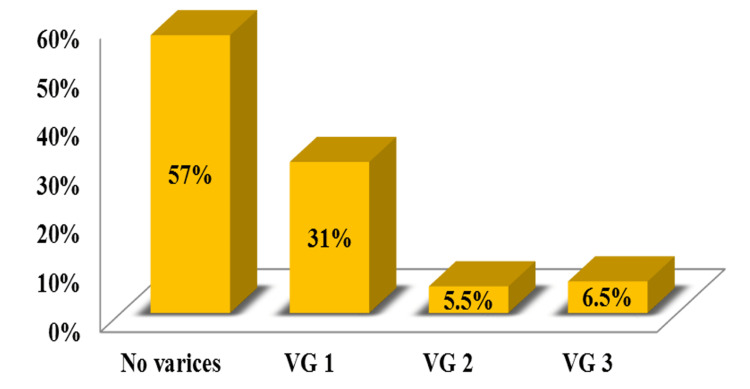
Distribution of varices grades among the study population VG: variceal grade

In our study population, 57% (n=96) had no varices, while 43% (n=72) had varices. Of the total 72 patients with varices, 52 patients (72.2%) had F1/Grade I varices, 9 patients (12.5%) had F2/Grade II varices, and 11 patients (15.3%) had F3/Grade III varices as shown in Figure 1.

We studied the ultrasound abdomen findings like liver size, echotexture, spleen size, portal vein size, splenic vein size, and the presence of ascites and their association with varices (Table 2).

**Table 2:** Ultrasound abdomen and their association with varices

Parameters	Varices n (%)		Total	p-value
	Absent	Present		
Liver echotexture				
Normal	21 (67.7%)	10 (32.3%)	31 (18.5%)	<0.001
Increased	58 (67.4%)	28 (32.6%)	86 (51.2%)	
Coarse	17 (33.3%)	34 (66.7%)	51 (30.3%)	
Total	96 (57%)	72 (43%)	168 (100%)	
Liver size				
Normal	89 (57%)	67 (43%)	156 (92.9%)	0.931
Abnormal	7 (58.3%)	5 (42.7%)	12 (7.1%)	
Total	96 (57%)	72 (43%)	168 (100%)	
Spleen size				
Normal	80 (67%)	39 (33%)	119 (70,8%)	<0.001
Splenomegaly	19 (33%)	33 (67%)	49 (29.2%)	
Total	96 (57%)	72 (43%)	168 (100%)	
Portal vein diameter				
Normal	93 (60.4%)	61 (39.6%)	154 (91.7%)	0.005
Abnormal	3 (21.4%)	11 (78.6%)	14 (8.3%)	
Total	96 (57%)	72 (43%)	168 (100%)	
Splenic vein diameter				
Normal	92 (58%)	67 (42%)	159 (94.6%)	0.429
Dilated ≥10 mm	4 (44.4%)	5 (55.6%)	9 (5.4%)	
Total	96 (57%)	72 (43%)	168 (100%)	
Ascites				
Absent	89 (63%)	53 (37%)	142 (84.5%)	0.001
Mild	7 (33.3%)	14 (66.7%)	21 (12.5%)	
Moderate	0 (0.0%)	5 (100%)	5 (3%)	
Total	96 (57%)	72 (43%)	168 (100%)	

Varices were found in 66.7% of patients with coarse echotexture of the liver, 32.6% of the patients with increased echotexture, and 32.3% of patients with normal echotexture. Of the patients with splenomegaly, 67% had varices and only 33% with normal spleen had varices. Varices were present in 78.6% of the patients with dilated portal veins (≥13 mm) or thrombosed portal veins, while only 39.6% of patients with normal portal veins had varices indicating a statistical significance (p-value -0.005). About 55.6% of patients with dilated splenic veins (≥10 mm) had varices, whereas 42% of the patients with normal splenic veins had varices that were not statistically significant (p-value -0.429). All the patients with moderate ascites (n=5) had varices, and 66.7% of the patients with mild ascites (n=14) and 37% of patients without ascites (n=53) had varices indicating a statistical significance (p-value =0.001). Statistical analysis was done for laboratory parameters like platelet counts, serum albumin levels, and albumin globulin reversal, and their association with esophageal varices is shown in Table 3.

**Table 3:** Laboratory parameters and their association with varices AG: albumin/globulin

Parameters	Varices n (%)		Total	p-value
	Absent	Present		
Platelet count (per microlitre of blood)				
<150000	34 (47.2%)	38 (52.8%)	72 (42.9%)	0.024
>150000	62 (64.6%)	34 (35.4%)	96 (57.1%)	
Total	96 (57%)	72 (43%)	168 (100%)	
Albumin (grams/decilitre)				
<=3.0	19 (39.6%)	29 (60.4%)	48 (28.6%)	0.001
3.1-3.5	22 (52.4%)	20 (47.6%)	42 (25%)	
>3.5	55 (70.5%)	23 (29.5%)	78 (46.4%)	
Total	96 (57%)	72 (43%)	168 (100%)	
AG reversal				
Present	40 (47.6%)	44 (52.4%)	84 (50%)	0.024
Absent	56 (66.7%)	28 (33.3%)	84 (50%)	
Total	96 (57%)	72 (43%)	168 (100%)	

In patients with platelet less than 150000 per microlitre of blood, 52.8% had varices with a statistically significant p-value of 0.024. In patients with low albumin (<3 g/dL), 60.4% had varices, 47.6% of patients with albumin between 3 and 3.5 g/dL had varices, and only 29.5% of those with albumin >3.5 g/dL had varices with a statistically significant p-value of 0.001. Among the patients with albumin globulin reversal, 52.4% had varices, while only 33% of those with normal albumin globulin ratio had varices that were significant with a p-value of 0.024. We also observed that as the liver elastography grading increases, the presence of varices also increases. Around 72.7% of patients with greater than F4 grade had varices, while 69.7% of those with F4 grade and 43% with F3 grade had varices. However, only a minority of the population with liver elastography F2 grade or below had varices. This association was statistically significant with a p-value of <0.001 (Table 4).

**Table 4:** Liver elasticity scores and their association with esophageal varices

Liver elasticity grade (kPa)	Varices		Total	p-value
	Absent	Present		
F0 (<5.1)	2 (100%)	0 (0.0%)	2 (1.1%)	<0.001
F1 (5.1-7.1)	22 (95.7%)	1 (4.3%)	23 (13.7%)	
F2 (7.2-9.1)	29 (83%)	6 (17%)	35 (20.8%)	
F3 (9.2-12.7)	24 (57%)	18 (43%)	42 (25%)	
F4 (12.8- 18.8)	10 (30.3%)	23 (69.7%)	33 (19.7%)	
>F4 (>18.8)	9 (27.3%)	24 (72.7%)	33 (19.7%)	
Total	96 (57%)	72 (43%)	168 (100%)	

Next, we studied scores like CTP scores, MELD scores, APRI scores, and FIB-4 in predicting the presence of esophageal varices (Table 5).

**Table 5:** Liver scores in predicting esophageal varices CTP: Child-Turcotte-Pugh, MELD: model for end-stage liver disease, APRI: aspartate aminotransferase-to-platelet ratio, FIB-4: fibrosis 4, AAR: aspartate aminotransferase-to-alanine aminotransferase ratio

Liver score	Varices n (%)			Total		p-value	
	Present	Absent					
1. CTP score							
CTP-A (5-6)	80 (64.5%)		44 (35.5%)		124 (73.8%)		0.003
CTP-B (7-9)	15 (38.5%)		24 (61.5%)		39 (23.2%)		
CTP-C (≥10)	1 (20%)		4 (80%)		5 (3%)		
Total	96 (57%)		72 (43%)		168 (100%)		
2. MELD score							
<11	67 (72%)		26 (28%)		93 (55.4%)		<0.001
>11	29 (38.7%)		46 (61.3%)		75 (44.6%)		
Total	96 (57%)		72 (43%)		168 (100%)		
3. APRI score							
<1	85 (60%)		57 (40%)		142 (84.5%)		0.096
>1	11 (42.3%)		15 (57.7%)		26 (15.5%)		
Total	96 (57%)		72 (43%)		168 (100%)		
4. FIB-4 score							
<1.45	50 (77%)		15 (23%)		65 (38.7%)		<0.001
1.46-3.25	34 (55.7%)		27 (44.3%)		61 (36.3%)		
>3.25	12 (28.6%)		30 (71.4%)		42 (25%)		
Total	96 (57%)		72 (43%)		168 (100%)		
5. AAR							
<0.8	15 (57.7%)		11 (42.3%)		26 (15.5%)		0.170
>0.8	81 (57%)		61 (43%)		142 (84.5%)		
Total	96 (57%)		72 (43%)		168 (100%)		

Varices were found in 80% of patients with CTP class C, 61.5% in those in CTP class B, and 35.5% with CTP class A. There was a significant association of CTP score with varices (p-value -0.003) indicating that higher CTP scores had more proportion of developing varices. On analysing the patients with MELD score, 61.3% had varices with a score greater than 11, while only 28% of patients with a MELD score less than 11 had varices indicating that a higher MELD score is associated with varices (p-value <0.001). A majority of the patients (57.7%) with APRI scores greater than or equal to 1 had varices, but only 40% of the patients with a score less than 1 had varices. There was no significant association between APRI scores and varices (p-value -0.096). In patients with FIB-4 scores more than 3.25, 71.4% had varices, 44.3% had varices with scores between 1.46 and 3.25, and 23% of patients with scores less than or equal to 1.45 had varices. This difference was statistically significant with a p-value of <0.001. Analysis of AAR scores revealed that 43% of the patients with an AAR of ≥0.8 had varices, while 42.3% of patients with an AAR of <0.8 had varices, and this association was not statistically significant (p-value -0.170).

Significant predictors of esophageal varices using binary logistic regression included patients beyond 60 years of age, those with conventional ultrasound abdomen features like coarse liver echotexture, splenomegaly (>12 cm), thrombosed or dilated portal vein (>13 mm), the presence of ascites, and those with thrombocytopenia <150000 per microlitre, albumin <3 g/dL, and reversal of albumin globulin ratio had significant odds of having varices. All these associations were statistically significant with a p-value less than 0.05 and a 95% CI above 1. The odds ratio for significant scores in predicting esophageal varices using binary logistic regression was significant in patients with a liver elastography grade more than F4, patients with a CTP-B score, patients with a MELD score of >11 and FIB-4 score of >3.25 and 1.46-3.25 (Table 6).

**Table 6:** Significant predictors of esophageal varices using binary logistic regression A/G: albumin/globulin, CTP: Child-Turcotte-Pugh, MELD: model for end-stage liver disease, FIB-4: fibrosis 4, CI: confidence interval

Factors	Categories	p-value	Odds ratio	95% CI	
				Lower limit	Upper limit
Age (years)	15-30	-	-	-	-
	31-45	0.466	1.6	0.42	6.72
	46-60	0.050	3.8	1.00	14.73
	>60	<0.001	14	3.26	66.12
Liver echotexture	Normal	-	1	-	-
	Increased	0.967	1.01	0.42	2.44
	Coarse	0.003	4.20	1.62	10.88
Spleen size	Normal	-	1	-	-
	Splenomegaly	<0.001	4.23	2.08	8.59
Portal vein	Normal	-	1	-	-
	Dilated/thrombosed	0.010	5.59	1.49	20.86
Ascites	Absent	-	1	-	-
	Present	0.001	4.56	1.79	11.56
Platelet (per microlitre of blood)	>150000	-	1	-	-
	<150000	0.025	2.04	1.09	3.80
Albumin (mg/dl)	>3.5	-	1	-	-
	3.1-3.5	0.028	2.17	1.00	4.78
	<=3.0	0.001	3.65	1.71	7.77
A/G reversal	Absent	-	1	-	-
	Present	0.013	2.2	1.18	4.10
Liver elastography scores	-	1	-	-
	>F4	<0.001	4.57	2.025	10.334
CTP score	A (5-6)	-	1	-	-
	B (7-9)	0.005	2.909	1.384	6.113
	C (> = 10)	0.080	7.273	0.788	67.09
MELD score	<11	-	1	-	-
	>11	<0.001	4.088	2.136	7.821
FIB-4 score	<1.45	-	-	-	-
	1.46-3.25	0.013	2.647	1.229	5.7
	>3.25	<0.001	8.33	3.443	20.167

**Figure 2:**
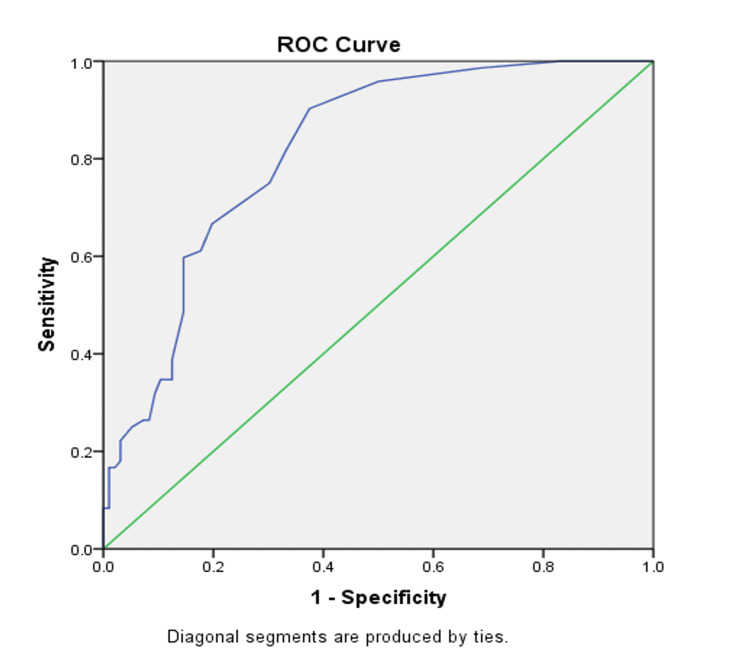
ROC curve for the liver elastography score in predicting esophageal varices ROC: receiver operating characteristic

The liver elastography scores were analysed for diagnostic accuracy using the receiver operating characteristic (ROC) curve for predicting varices as shown in Figure 2.

**Figure 3:**
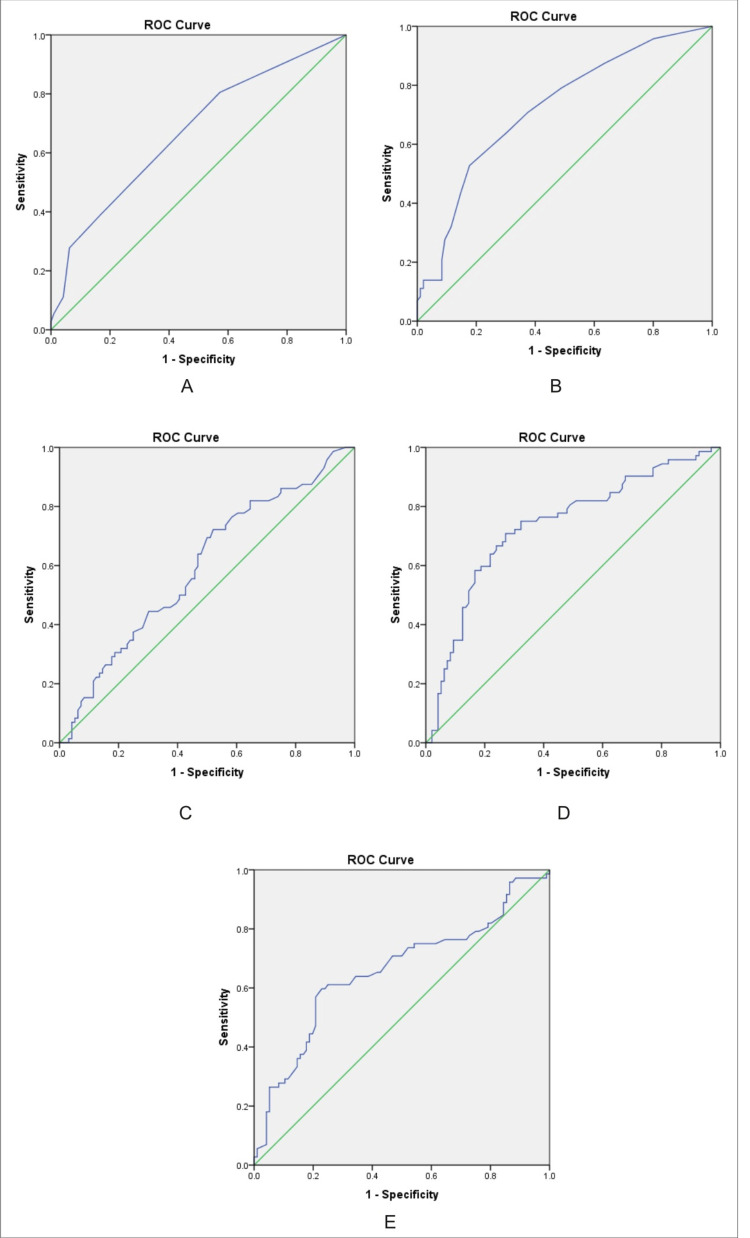
ROC curve for the different scores used in predicting esophageal varices ROC: receiver operating characteristic. A: CTP score. B: MELD score. C: APRI score. D: FIB-4 score. E: AAR

The area under the curve was 0.817 which implies that 81.7% is the diagnostic accuracy of the liver elastography score that was statistically significant (p <0.001). ROC curve for the CTP scores (67%), MELD scores (72%), APRI scores (59.5%), FIB-4 scores (73.6%), and AAR (65.8%) in predicting varices was done. The diagnostic accuracy of all the scores was statistically significant with p <0.05 and CIs below 1 (Figure 3).

**Figure 4:**
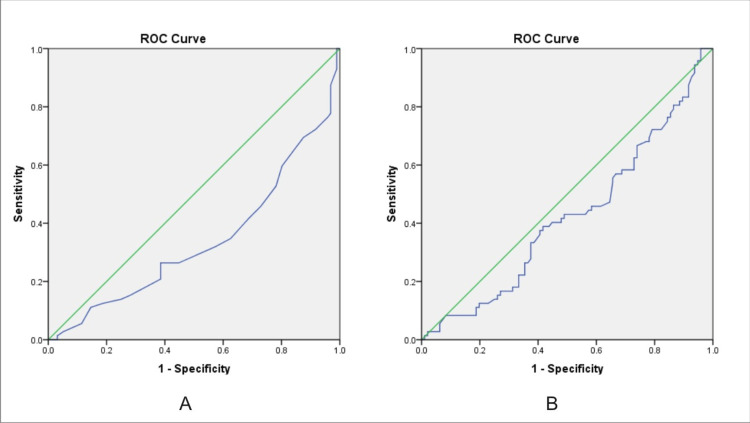
ROC curve for albumin and platelet in predicting esophageal varices ROC: receiver operating characteristic. A: albumin. B: platelets

The albumin values were analysed using the ROC curve with a diagnostic accuracy of 33.7% which was statistically significant (p-value <0.001). The platelet values were analysed using the ROC curve with a diagnostic accuracy of 42.3% but were not statistically significant (p-value -0.087) (Figure 4).

## Discussion

Patients with chronic liver disease are routinely screened for varices, and it has been observed that many patients do not have varices or have only mild varices that do not require intervention. Our study is facilitated in a way that the non-invasive predictors of varices in chronic liver disease will reduce the burden of endoscopy for both the patient and the physician and also in areas where endoscopic procedures are not readily available. Here, we studied liver elastography as a predictor of esophageal varices and compared it with other non-invasive predictors like ultrasound abdomen and liver function tests. The median age of 48 years and male gender predominance observed in our study was comparable with the study done by Cherian et al. [9], in which varices were found in the majority in the age groups ranging from 46 to 60 years and male gender prevalence of 73.2%. We observed a high prevalence of diabetes and hypertension among the patients with varices. Alcohol was the leading cause of cirrhosis in this study followed by nonalcoholic steatohepatitis (NASH), hepatitis B, and hepatitis C infection which was comparable to the study by Sharma et al. [12] and unlike the study by Bota et al. [13] where hepatitis C was the commonest cause. In this study, 31% of the subjects had Grade I varices followed by 5.5% and 6.5% with Grade II varices and Grade III varices, respectively, which was comparable to other similar studies [9,12].

Various other studies revealed that changes in the echogenicity of the liver surface and the presence of nodularity correlated well with the severity of fibrosis and cirrhosis [14-16] which was comparable in our study. However, the binary logistic regression showed only the association between coarse echotexture and varices as significant. In our study, the altered liver size (both shrunken liver <10 cm and enlarged liver >16 cm) did not have a significant association with the presence of varices unlike the study by Sharma et al. [12] which proved the liver size influenced the presence of varices. Our study predicted a greater statistical association of varices (67%) with splenomegaly >12 cm which was in agreement with several other studies [9,16,17]. Similarly, the association of varices with dilated or thrombosed portal vein was statistically significant in our study and was comparable with similar studies by Cherian et al. [9] and Mandal et al. [17]. However, there was an insignificant association of varices with the splenic vein diameter. Sharma et al. [12] in his study proved that there was an insignificant association between varices and the size of the portal vein or splenic vein diameter. In our study, 100% of patients with moderate ascites had varices, 66.7% with mild ascites had varices, and 37% without ascites had varices which were similar to other studies [18,19].

Our study showed a significant association between platelet count and prediction of esophageal varices with an odds ratio of 2.04 (95% CI 1.09-3.80). The ROC curve for the platelets in predicting varices showed 79000 per microlitre of blood as cut-off with 90.3% sensitivity. However, there was no statistical significance when the study population was divided into two groups based on the 80,000 per microlitre of blood cut-off. The ROC curve for the albumin level in predicting esophageal varices showed 2.25 as the cut-off value with 90.3% sensitivity. The area under the curve was 0.337 which was statistically significant (p <0.001). The studies by Cherian et al. [9], Sharma et al. [12], and Ng et al. [20] proved a significant association between platelet count and the prediction of esophageal varices but did not find a statistically significant association between albumin values and esophageal varices. We also found that the occurrence of varices increased as the elastography grade increased. The ROC curve for liver elastography score in predicting esophageal score showed >11.5 as the best cut-off with 75% sensitivity, 70% specificity, and 76% negative predictive value. Bota et al. [13] used ARFI measurements similar to our study and found combining spleen stiffness and liver stiffness would be a better predictor of varices rather than using liver stiffness alone.

In this study, a greater proportion of varices was seen in patients with CTP class B (61.5%) and CTP class C (80% ). Logistic regression showed a significant association of esophageal varices with class B but not with class C. This difference could be due to the very less number of patients in class C (n=5). A similar study by Cherian et al. [9] concluded that CTP scoring is a significant individual predictor of esophageal varices. In this study, a MELD score of >11 was associated with the presence of varices and was significant. The ROC curve for the MELD score in predicting esophageal varices showed a cut-off of 9.5 with 71% sensitivity and 63% specificity. MELD scores offered an objective and accurate prognostic prediction as established by various studies by Wang et al. [21] and Reverter et al. [2]. Our study also demonstrated a significant association between FIB-4 score and varices. About 71.4% of patients with FIB-4 scores above 3.25 had varices with 8.3 times higher odds of having varices than the less than 1.45 score. This was comparable to the meta-analysis done by Deng et al. [11] revealed that the FIB-4 score had a pooled sensitivity of 62% in predicting large varices. In our study, the association of APRI with esophageal varices was studied by using two different cut-offs (0.7 and 1), and there was no significant association in both types even though a greater proportion of patients with an APRI score of >0.7 (51%) and those with an APRI score of >1 (57.7%) had varices.

Limitations of our study

The association of multiple comorbidities in a single patient and their effect on the occurrence of esophageal varices were not studied. The different grades of varices in the different groups of the study population were not predicted. Liver elastography was not repeated after the normalisation of aminotransferase levels. Hence, the effect of aminotransferase elevation on the alteration of liver elasticity values and other scores like AAR, APRI, and FIB-4 scores was not studied. The majority of the studies were done using a Siemens machine using an ARFI or Fibro scan using the transient elastography technique, while we used Toshiba Aplio 500 ultrasound 2D-SWE using ARFI. The liver elasticity values were not comparable since each machine has its own standardised set of values for different grades of fibrosis. Spleen stiffness measurement along with liver stiffness is found to be a better predictor of esophageal varices, but in our study, splenic stiffness was not measured.

## Conclusions

Liver elastography is a non-invasive procedure that can be a useful tool in predicting esophageal varices in chronic liver disease patients even though the gold standard is upper GI endoscopy. Clinicians should be able to predict the risk of esophageal varices using non-invasive methods like laboratory parameters, ultrasound findings, and liver elastography in patients with chronic liver disease and should intervene early to avoid unfavourable outcomes. Several such studies are required on a larger population to understand the impact of non-invasive modalities in predicting the risk of variceal bleeding so that it can be a replacement for invasive procedures for patients.
